# P353: The problem of patient safety in the provinces of Maniema and south Kivu in the Democratic Republic of Congo

**DOI:** 10.1186/2047-2994-2-S1-P353

**Published:** 2013-06-20

**Authors:** AK Mwadi, SEM Bdom, M Kavul, M Selua, A Ajfma

**Affiliations:** 1 RIPAQS-DRC, Kinshasa, Congo, The Democratic Republic of the

## Introduction

Patient safety is now a global public health priority. In DR Congo, after several years of armed conflict in particular in the East, it is imperative to know the inventory of patient safety in a post-conflict situation in the country.

## Objectives

To evaluate the perception and arrangements on patient safety by personal health care facilities in the eastern DR Congo.

## Methods

Using a standardized questionnaire, interviews, focus groups and field visits were conducted with ten health centers randomly selected Uvira and Kindu in the provinces of Maniema and South Kivu.

Items focused on the organization of health, perception of patient safety and prevention of risk. The observation concerned the operating rooms, maternity wards, labs, sterilization and waste treatment.

## Results

It was found: In knowledge: 80% interviewees are aware of the risks of nosocomial infections and 90% are not aware about the safety of patients, 62.5% believe that it is not a priority given insufficient resources, 100% believe that it is the responsibility of government authorities. Plan comments: No incinerator in 100% of the 10 facilities visited, Using disinfecting means: bleach as a disinfectant (20%), soap powder (80%) Lack of piped water (100 %) in operating rooms, care and childbirth; Presence sterilizing equipment: poupinel oven (10%), charcoal autoclave (10%), lack of sterilization procedures (100%).

## Conclusion

The safety of patients is not known and neither a major concern in the structures hosting visited sick and family members who make long journeys in search of care.

## Disclosure of interest

None declared

